# A Microchip for High-Throughput Axon Growth Drug Screening

**DOI:** 10.3390/mi7070114

**Published:** 2016-07-07

**Authors:** Hyun Soo Kim, Sehoon Jeong, Chiwan Koo, Arum Han, Jaewon Park

**Affiliations:** 1 Department of Electrical and Computer Engineering, Texas A&M University, College Station, TX 77843, USA; hs81.kim@gmail.com (H.S.K.); arum.han@ece.tamu.edu (A.H.); 2 Department of Biomedical Engineering, Texas A&M University, College Station, TX 77843, USA; sehoon.jeong@tamu.edu; 3 Department of Electronics and Control Engineering, Hanbat National University, Daejeon 305-719, Korea; cwankoo@hanbat.ac.kr; 4 Department of Electrical and Electronic Engineering, Neural and Cognitive Sciences Research Center, Southern University of Science and Technology, Shenzhen 518055, China

**Keywords:** microfluidic neuron culture platform, compartmentalized culture, localized biomolecular treatment, high-throughput screening

## Abstract

It has been recently known that not only the presence of inhibitory molecules associated with myelin but also the reduced growth capability of the axons limit mature central nervous system (CNS) axonal regeneration after injury. Conventional axon growth studies are typically conducted using multi-well cell culture plates that are very difficult to use for investigating localized effects of drugs and limited to low throughput. Unfortunately, there is currently no other in vitro tool that allows investigating localized axonal responses to biomolecules in high-throughput for screening potential drugs that might promote axonal growth. We have developed a compartmentalized neuron culture platform enabling localized biomolecular treatments in parallel to axons that are physically and fluidically isolated from their neuronal somata. The 24 axon compartments in the developed platform are designed to perform four sets of six different localized biomolecular treatments simultaneously on a single device. In addition, the novel microfluidic configuration allows culture medium of 24 axon compartments to be replenished altogether by a single aspiration process, making high-throughput drug screening a reality.

## 1. Introduction

Damages in central nervous system (CNS) often result in permanent deficit and it has been long thought that adult mammalian CNS cannot be functionally recovered after injury, unlike peripheral nervous system (PNS). Major factors assumed to inhibit the CNS axons regeneration or self-recovery after damage were physical/chemical extrinsic environmental factors such as formation of glia scars around the damaged axons and myelin-associated inhibitors (e.g., Myelin-associated glycoprotein (MAG), oligodendrocyte myelin glycoprotein (OMgp), or Nogo) [1,2]. However, some of recent research has shown that manipulation of axonal intrinsic growth control pathways promotes CNS axon regeneration while neutralization of commonly known axon inhibiting extrinsic factors results in only limited axon regeneration in vivo [3,4,5]. This clearly indicates that both the extrinsic inhibitory factors as well as the intrinsic growth capability of the neurons contribute to the regeneration of CNS axons.

In order to understand the relationship between the intrinsic growth capability and the regeneration mechanism as well as to discover potential growth factors or drugs that promote CNS axon growth/regeneration, an in vitro tool that enables precise and localized chemical microenvironments control with higher efficiency is critically needed. Unfortunately, conventional in vitro axon regeneration/growth analysis studies that are typically carried out on multi-well cell culture plates hold several limitations in studying intrinsic growth capability of CNS axons. First, neuronal cell bodies and network of axons are exposed to a single culture medium bath. Therefore, chemical treatments affect both neuronal somata and axons simultaneously, hence, making it difficult to analyze the influence of chemical treatments on axons independently. In addition, secretion factors from neuronal somata directly affect their axons. In reality, many axons are apart from neuronal cell bodies in vivo and axons are often exposed to different environmental surroundings compared to the somata. Second, axons usually grow in random directions and tangle intensively with other axons and dendrites, which makes it almost impossible to quantitatively analyze the growth. Therefore, the studies are often performed at very low cell culture density (3–20 cells/mm^2^) and for only a very short period of time (<5 days) to track and to quantify the axons [6,7]. Considering that most in vitro neuron cultures are optimized at areal cell density of 250–1500 cells/mm^2^ [8,9,10,11], longer culture period at proper cell density might give more accurate results in vitro. 

Campenot chamber is probably the only conventional tool that isolates axons from neuronal somata for localized manipulation to axons [12]. Although it has been widely used for studying axon–glia interactions, the chamber not only requires complicated and labor intensive preparation steps but also shows low reliability due to fluid leakage from imperfect grease seal [13,14]. Therefore, a neuron culture platform capable of localized biomolecular treatments with higher throughput that can culture neurons at commonly used cell culture densities will be a powerful tool for studying in vitro CNS axon growth toward screening of potential drugs that can promote regeneration/growth. In 2005, Taylor et al. introduced a microfluidic based compartmentalized neuron culture platform made of poly(dimethylsiloxane) (PDMS) that mimics the conventional Campenot chamber [15]. Since then, microdevices utilizing similar configurations have been actively introduced for studying various neuroscience applications [16,17,18,19] that include axon regeneration studies where axonal regeneration followed by either chemical (e.g., biomolecular treatment to isolated axons) or physical (e.g., direct damage to isolated axons with integrated microfluidic components such as pneumatically actuated valves) axotomy can be investigated [20,21,22,23,24,25]. Although successful in performing localized axonal damage and demonstrating their potential use for axon regeneration studies, they were low in throughput and had limitations in practically screening potential drug candidates. Here, we present a PDMS high-throughput compartmentalized neuron culture platform having 24 axon isolation compartments, allowing four sets of six independent localized biomolecular treatments to be carried out in parallel. In addition, the novel microfluidic design of the device minimizes direct labor input toward a real high-throughput screening, where an only single aspiration step can replenish culture medium or biomolecular treatments of 24 compartments altogether.

## 2. Materials and Methods

### 2.1. Design

The conceptual framework of the proposed platform is based on our previous publication [26], where the soma compartment is connected to the satellite axon compartments via shallow microchannels, which confine neuronal somata to the soma compartments while enabling axons to pass through the microchannels into the axon compartments for isolation. Isolated axons can then be locally treated with various biomolecules in the absence of neuronal somata or dendrites for analyzing the effects of treatment on axonal growth. This not only enables localized biomolecular treatments to axons but also allows isolated axons to be quantitatively analyzed even at commonly used in vitro cell culture densities by measuring the area of the axon compartment covered with axons [27]. In order to increase the throughput of the device and to make it more practical for neuroscientists to use in their biology laboratories, we have designed the device to have 24 axon compartments, which has four times higher throughput compared to our previous work. However, designing a compartmentalized neuron culture platform with 24 compartments required more than simply extending the number of the satellite compartments. Merely increasing the number of compartments with previously used configuration requires tens of manual pipetting steps on a small (8 × 2.5 mm^2^) compartment when changing culture medium or applying biomolecular treatments, which can be very challenging and labor intensive. In addition, integrating multiple small-sized open compartments has potential high risk of cross-contamination of biomolecular treatments among neighboring axon compartments. Considering that culture medium needs to be replenished once every 3–4 days in these microdevices, manual pipetting steps can be a critical bottleneck in utilizing such multi-compartment culture platforms as a high-throughput screening tool. Out of 24 compartments, we have grouped four axon compartments into one experimental condition set that can be used as a four replicates of one biomolecular treatment. Since most biological experiments require 3–4 replicates for each condition being tested, the device is capable of conducting four sets of six independent experimental conditions on a single device simultaneously.

**Figure 1:**
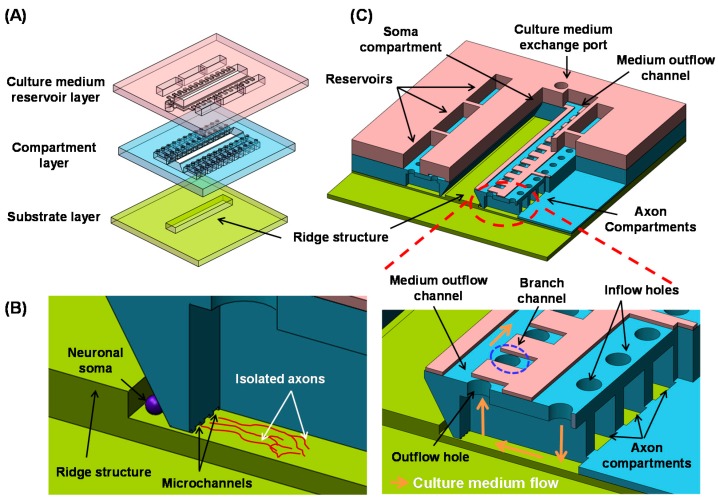
Illustrations of the high-throughput compartmentalized neuron culture platform: (**A**) three layers (i.e., culture medium reservoir layer, compartment layer with two through-holes at each axon compartment, cell culture substrate layer with the ridge structure) composing the platform; (**B**) an illustration showing the axon isolation from the neuronal somata by the shallow height of the microchannels connecting the soma compartment and the axon compartment; and (**C**) schematic illustrations of the assembled device showing cross-sections. Inset: A close-up view of the axon compartments showing the fluidic flow during the one-step culture medium replenishment process. Blue dotted circle indicates a branch channel that connects the outflow hole with the medium outflow channel.

The developed device is composed of three layers: a PDMS culture medium reservoir layer, a PDMS compartment layer, and a glass cell culture substrate layer with a PDMS ridge structure. The schematic illustration of the high-throughput compartmentalized neuron culture platform is shown in Figure 1A. The ridge structure is permanently bonded on the bottom glass substrate (50 × 50 mm^2^) and designed to be slightly smaller (7 × 38 mm^2^) than the size of the center soma compartment (10 × 40 mm^2^). The ridge structure has two features: (1) it reduces the size of cell culture area on the substrate and minimizes the number of cells required per device even at optimized cell culture density (1000 cell/mm^2^); and (2) the ridge structure allows neuronal cells plated to the soma compartment to be positioned closer to the array of microchannels. Since the axons grow in random directions, shorter distance of neuronal cell bodies from the microchannels results in increased axon crossing efficiency [27]. The compartment layer is composed of one rectangular soma compartment (10 × 40 mm^2^) in the center and 12 satellite axon compartments (2.4 × 8 mm^2^) on each side. The soma compartment and the axon compartments are connected via arrays of shallow microchannels (height: 3 μm, width: 20 μm, length: 400 μm) that confine neuronal somata only to the soma compartment while allowing axons to pass through. This feature enables isolation of axons for localized biomolecular treatments (Figure 1B). Each axon compartment is connected with the soma compartment by approximately 20 microchannels, which is enough for dense network of axons to be isolated inside the axon compartments. While the soma compartment is an open-compartment configuration, the axon compartments are semi-open configuration with two through-holes (diameter: 1.5 mm) defined on the ceiling of the each compartment (Figure 1C). These holes are used for one-step culture medium replenishment. One hole at the outer side is used for the inflow of the culture medium and is directly connected to the culture medium reservoir. The other hole at the inner side is used for the outflow of the depleted culture medium and is connected to the medium exchange port via the culture medium outflow channel patterned on the bottom side of the reservoir layer. In order to minimize the fluidic flow differences among 24 axon compartments during the one-step replenishment process, the branch channels (Figure 1C) that connect the medium outflow channel and the outflow holes have been designed to have the similar fluidic resistance for all 24 outflow holes. The culture medium reservoir layer is composed of six open reservoirs for the axon compartments and one for the soma compartment. Upon assembly of the reservoir layer with the compartment layer, inflow holes of the four axon compartments are positioned beneath one reservoir, representing one set of experiment conditions with four replicates, and the culture medium outflow channel connects all outflow holes with the exchange port. Using this design, negative pressure applied at the exchange port drives the new culture medium with added biomolecular treatments inside the six reservoirs into the 24 axon compartments simultaneously while aspirating out the nutrient depleted culture medium inside the axon compartments for disposal (Figure 1C orange arrows). With this novel design, six different localized biomolecular treatments on isolated axons with four replicates could be implemented on a single device with minimal pipetting steps towards potential automation.

### 2.2. Fabrication

**Figure 2:**
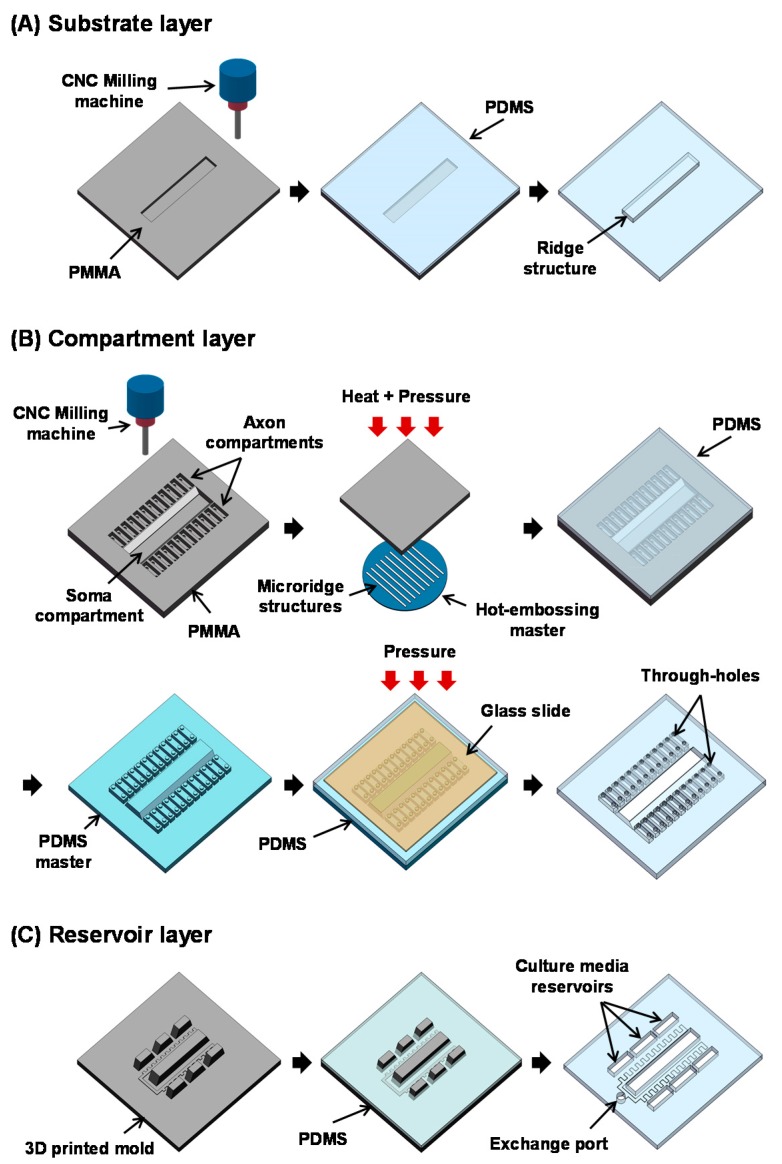
Fabrication processes of the high-throughput compartmentalized neuron culture platform; (**A**) substrate layer, (**B**) compartment layer, (**C**) reservoir layer.

The substrate layer was prepared by permanently bonding the ridge structure at the center of the glass cell culture substrate (Figure 2A). The PDMS ridge structure was fabricated by cast molding from a poly(methyl methacrylate) (PMMA) master mold engraved by the computer numerically controlled (CNC) milling machine (MDX 40, Roland, Irvine, CA, USA). The ridge structure was cut out after the PDMS softlithography and was treated with oxygen plasma (Harrick Plasma, Ithaca, NY, USA) for 2 min before bonding.

The compartment layer that incorporates 24 axon compartments and one soma compartment was fabricated by series of microfabrication processes including the micro-macro hybrid softlithography master fabrication method (MMHSM) [28], in order to manufacture a PDMS layer with pre-defined through holes, macroscale structures (i.e., compartments), and microstructures (i.e., microchannels) (Figure 2B). First, both the soma compartment and the axon compartment, structures were engraved on a PMMA by the CNC milling machine. Microchannel patterns were then transferred on the PMMA block with the compartment structures by a hot-embossing. The hot-embossing process was conducted at 115 °C with 1082 kPa of pressure for 5 min using a temperature controlled hydraulic press (Specac Ltd., London, UK). The hot embossing master, which has an array of 3 µm high and 20 µm wide microridge structures, was fabricated by anisotropically etching a silicon wafer in a 40% KOH solution at 60 °C [29]. A silicon nitride layer patterned by photolithography and RIE processes was used as the etch mask during the silicon etching process. The silicon nitride etch mask was patterned at a 45° angle to the primary flat of the (100) wafer, resulting in microridge structures with vertical side walls when the silicon was etched in KOH solution. The final PDMS compartment layer was fabricated by the double PDMS softlithography process from this PMMA master. First, a PDMS master was cast molded from the PMMA master followed by 15 min of vapor coating with (tridecafluoro-1,1,2,2-tetrahydrooctyl) trichlorosilane (United Chemical Technologies, Inc., Bristol, PA, USA) and briefly rinsed with isopropyl alcohol (IPA). Then, the PDMS compartment layer was replicated from the PDMS master. Through-holes for the culture medium inflow/outflow at the top of the axon compartments were obtained by covering the PDMS master with a glass slide coated with (tridecafluoro-1,1,2,2-tetrahydrooctyl) trichlorosilane and applying constant pressure (0.43 kPa) during the PDMS polymerization process. The applied pressure pushed away uncured PDMS between the PDMS master and the top glass slide, allowing a tight contact between them [30].

The PDMS culture medium reservoir layer was cast molded from a master prepared by a 3D printer (MINI, envisionTEC, Dearborn, MI, USA) using HTM140 as the resin material. The culture medium exchange port was manually punched out with a biopsy punch (Acuderm Inc., Fort Lauderdale, FL, USA). The reservoir layer, the compartment layer, and the substrate layer with the ridge structure were then treated with oxygen plasma and assembled together with alignment marks in each layer. Assembled devices were immersed in deionized (DI) water and degassed inside a vacuum chamber to ensure all channels and compartments are free of bubbles followed by sterilization in an autoclave. Detailed device dimensions are illustrated in Supplementary Figure S1.

### 2.3. Tissue Dissociation and Cell Preparation

Primary CNS neurons used in the manuscript were prepared from forebrains of embryonic day 16 (E16) Sprague-Dawley rats [31]. Forebrains free of meninges were dissected in ice-cold dissection buffer (Ca^2+^/Mg^2+^-free Hank’s Balanced Salt Solution containing 10 mM HEPES) (Invitrogen, Carlsbad, CA, USA), dissociated with L-cysteine activated papain (10 units/mL) (Sigma Aldrich, St. Louis, MO, USA) for 5 min at 37 °C, and resuspended in dissection medium containing trypsin inhibitor (10 mg/mL) (Invitrogen, Carlsbad, CA, USA) for 2–3 min followed by washing with the trypsin inhibitor solution. The tissue was resuspended in a plating medium (NBB27 + glutamate: neurobasal medium containing 2% B27, 1 mM Glutamine, 25 μM glutamic acid, 100 units/mL penicillin, and 100 μg/mL streptomycin) (Invitrogen, Carlsbad, CA, USA) and triturated with a fire-polished glass Pasteur pipette. The cells were then passed through a 70 μm cell sieves and live cells were counted using a hemocytometer and trypan blue exclusion assay.

### 2.4. Microchip Cell Culture

Sterilized devices were coated with poly-d-lysine for 24 h at room temperature followed by rinsing with DI water. Neurons prepared from E16 rats were diluted in the 300 μL of plating medium (NBB27 with glutamate) and loaded into the soma compartment at an areal density of 1000 cells/mm^2^. Culture medium (150 μL) was also added to each axon reservoir to prevent the substrate from drying. The device loaded with neurons was stored inside a 37 °C humidified 5% CO_2_ incubator for 30 min for allowing cells to settle down and attach to the substrate layer. After the 30 min incubation, 600 μL and 200 μL of culture medium was added to the soma compartment and to each axon reservoirs, respectively. Cells were then cultured inside a 37 °C humidified 5% CO_2_ incubator without culture medium change for four days. Culture medium was replaced with NBB27 without glutamate at four days in vitro (DIV 4) and was exchanged every 3–4 days.

### 2.5. Localized Biomolecular Treatment

Fluidic isolation between the center soma compartment and the axon compartments was achieved by the fluidic level difference. During the localized biomolecular treatment to the isolated axons, the soma compartment is set to have slightly higher fluidic level compared to the axon compartments, thereby preventing biomolecules added into the axon compartments from diffusing or flowing into the soma compartment. The concept has been well established by us as well as others [15,32,33,34,35,36,37]. In addition, in order to demonstrate that the device is capable of performing six different biomolecular treatments in a single device in parallel, 5 µg/mL of chondroitin sulfate proteoglycan (CSPG) (Millipore, Billerica, MA, USA), known to cause degeneration of axons, were added to the two reservoirs in the middle [38,39].

### 2.6. Imaging

Cells cultured inside the device were stained with Calcein-acetoxymethyl (AM) (Sigma Aldrich, St. Louis, MO, USA) for visualization and imaged with a fluorescent microscope (IX71, Olympus, Center Valley, PA, USA) equipped with a camera (DP70, Olympus). First, cells were briefly rinsed with a phosphate buffered saline (PBS) and 1 μM of Calcein-AM was added to both the soma compartment and the axon compartments followed by incubation in a 37 °C humidified incubator for 10 min. Cells were rinsed with PBS three more times before acquiring images.

### 2.7. Fluidic Simulation

Dimensions (i.e., height and width) of all branch channels connecting the culture medium outflow channel and the outflow holes in each axon compartment has been optimized using a commercially available finite element method (FEM) software (COMSOL Multiphysics^®^, COMSOL Inc., Los Angeles, CA, USA) for minimizing the fluidic resistance difference. Flow profiles of culture medium across the outflow channel were analyzed under a negative pressure applied condition (i.e., during the culture medium replenishment process).

## 3. Results and Discussion

**Figure 3:**
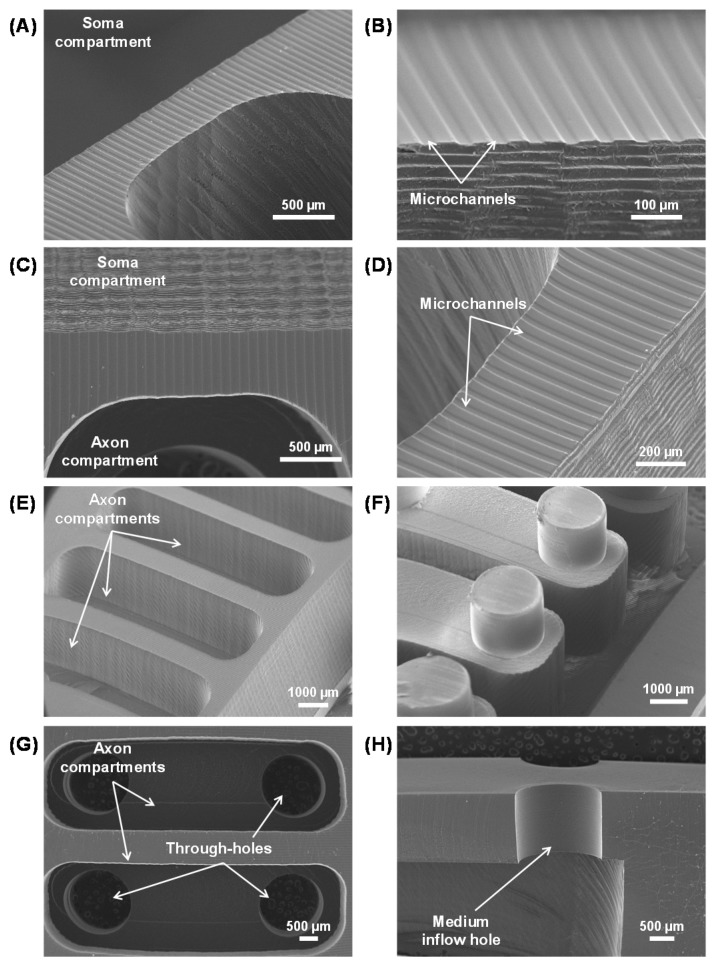
SEM images of the fabricated devices: (**A**,**B**) PMMA master showing precisely patterned microchannel array by the hot-embossing process; (**C**–**E**) bottom-side of the final PDMS layer showing 3 μm deep and 20 μm wide microchannels connecting the soma compartment and the axon compartments; (**F**) axon compartment of the PDMS master, in which the round pillar-like structures allows pre-defined holes to be made on the final PDMS layer; and (**G**,**H**) pre-defined through-holes on the axon compartments of the final PDMS layer.

The compartment layer was prepared by integrating multiple microfabrication processes (e.g., hot-embossing, MMHSM, double PDMS softlithography, and sandwich PDMS polymerization). Therefore, reliability of the processes and the accuracy of the fabricated master molds as well as the PDMS replica were investigated. Figure 3A,B shows the scanning electron microscope (SEM) images of the PMMA master after the hot-embossing process. It can be clearly seen that 3 μm deep and 20 μm wide microchannel structures are precisely transferred to the PMMA master and are connecting the center soma compartment with 24 satellite axon compartments. Images of final PDMS replica are shown in Figure 3C–E, where no significant changes in structures and dimensions had been made during the multiple replication processes. Each axon compartment has two through-holes that are 1.5 mm in diameter for culture medium inflow and outflow. Holes on PDMS layers are most commonly defined by manually punching it with a biopsy punch; however, punching a total of 48 small holes on a single device is a labor intensive and an inaccurate process. The fabrication method used here resulted in the compartment layer with accurately and cleanly cut through-holes by a single PDMS replication process. Elastic property of the PDMS and the constant pressure ensured a tight contact between the pillar-like structures of the PDMS master (Figure 3F) with the glass slide during the polymerization and enabled to fabricate a PDMS replicate with pre-defined holes (Figure 3G,H), ruling out a time-consuming manual punching process.

**Figure 4:**
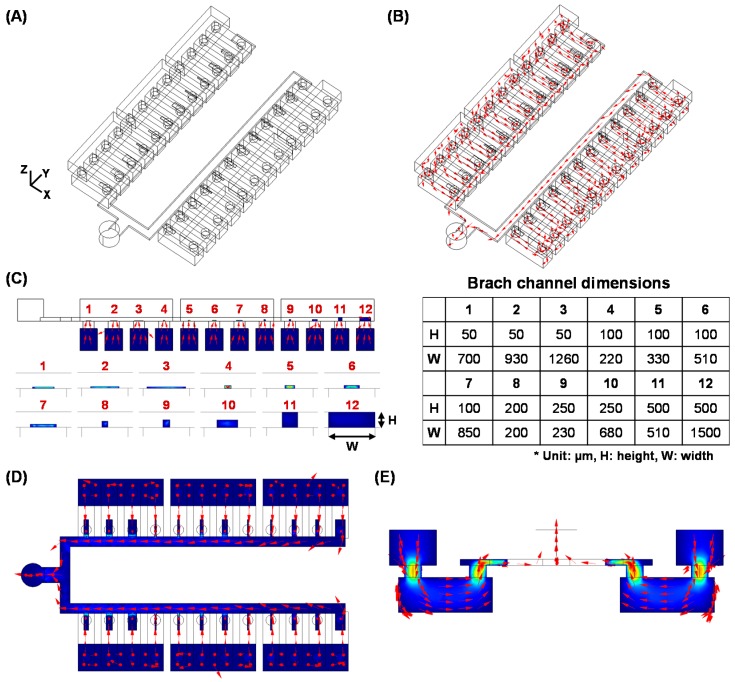
Numerical simulation results of the culture medium flow profiles during the replenishment process. (**A**) 3D illustration of the culture medium outflow channel connected to the axon compartments and the reservoirs as well as the culture medium exchange port. (**B**) Normalized culture medium flow streamline showing the flow direction from the reservoirs to the culture medium exchange port. (**C**) *y*-*z* cross-sectional view at the center of branch channel part of which enlarged views show the design used for matching the fluidic resistance at each axon compartments. Table illustrates the dimensions of each branch channel used. (**D**) *x*-*y* cross-sectional view at the culture medium outflow channel. (**E**) *x*-*z* cross-sectional view across the center of 12th branch channel part. Both (D) and (E) clearly show the culture medium flow profile collected at the medium exchange port through axon compartments and the culture medium outflow channel under a negative pressure applied condition.

To achieve efficient medium replenishment, branch channel dimensions and fluidic flow profiles during the replenishment process were optimized and analyzed through numerical simulation (Figure 4A). The fluidic resistances between each axon compartment and the culture medium exchange port have to be similar so that the nutrient depleted medium from all 24 compartments can be simultaneously replaced within a short period of time. As shown in Figure 4, different dimensions (i.e., width and height) of the branch channels that interconnect the culture medium outflow channel and the outflow holes in each axon compartment were designed to compensate the fluidic resistance differences among the compartments [40]. One aspect to note here is that five different heights (50, 100, 200, 250, and 500 μm) in the branch channel designs were selected based on the resolution of the 3D printer (25 μm) used for the master fabrication. First, flow streamlines of the culture medium from each axon compartment to the culture medium exchange port were analyzed. When negative pressure is applied, the force drives nutrient depleted medium inside the axon compartments towards the exchange port while allowing new culture medium inside the reservoirs to move into the axon compartments (Figure 4B–E). In addition, this negative pressure prevents the extracted depleted medium from flowing back into other neighboring axon compartments, enabling the fluidic isolation among axon compartments (Figure 4B,D,E). Since the culture medium outflow channel has a symmetric structure, where opposite branch channels on both sides comprise the same dimensions, a symmetric culture medium flow profile was observed throughout the channel design. Next, the flow rates across the branch channels at each axon compartment were characterized. As seen in Figure 4C and Supplementary Figure S2, similar flow rates were observed for each branch channel where culture medium passed with different flow speed (visualized with different color spectrum, red: faster speed, blue: slower speed), resulting from the adjusted fluidic resistance with different channel dimensions. To further confirm the effect of the compensated fluidic resistance, the flow profile of a device having identical dimension branch channels (height: 500 μm, width: 1500 μm) was analyzed and compared to our current design (Supplementary Figure S2). While relatively uniform fluidic flow was observed at each branch channel in the current design, the design without the compensation showed uneven fluidic flow where most of flow was found from the axon compartments closer to the culture medium exchange port. The flow profile difference shows the effectiveness of the novel microfluidic configuration that incorporates the fluidic resistance compensating branch channels in the platform.

**Figure 5:**
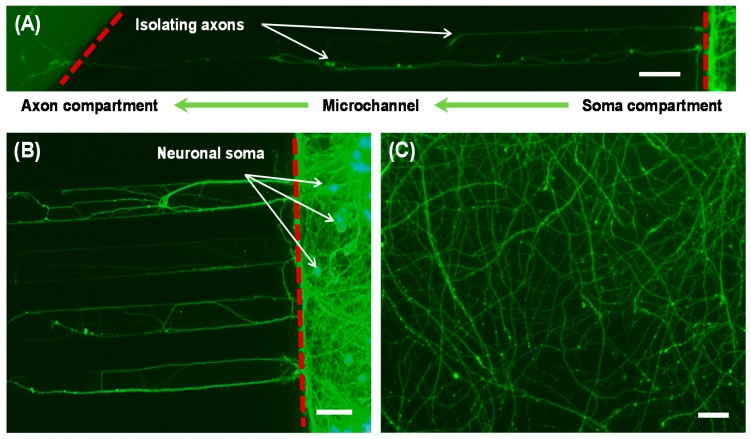
(**A**,**B**) Axons being isolated from the neuronal somata by the shallow microchannels connecting the soma compartment and the axon compartment. Red dotted lines indicate compartment boundaries. (**C**) Isolated axonal layer inside the soma compartment at DIV 12. Cells were stained with Calcein-AM for visualization. Scale bars: 50 µm.

Neurons from E16 rats were added into the soma compartment and cultured up to 12 days in vitro and axons successfully differentiated from neuronal somata and passed through the microchannel arrays into the neighboring axon compartment (Figure 5A). The shallow height of the microchannels confined neuronal somata to the soma compartment and the ridge structure allowed somata to position close to microchannels, resulting in dense isolated axonal network formation at DIV 12 (Figure 5B,C).

**Figure 6:**
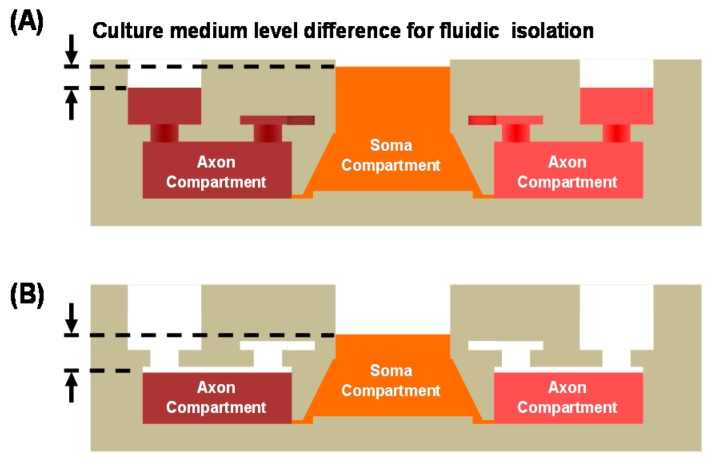
Cross-sections of the high-throughput compartmentalized neuron culture platform showing the fluidic isolation scheme during: (**A**) the culture medium replenishment process; and (**B**) localized biomolecular treatment.

**Figure 7:**
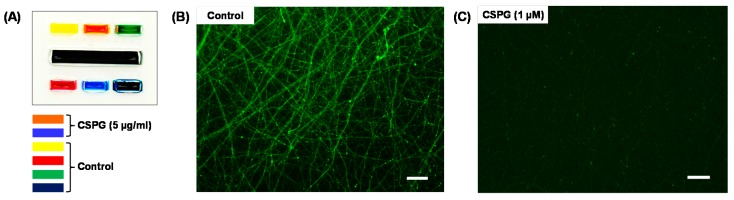
(**A**) Image of the high-throughput compartmentalized neuron culture platform with reservoirs filled with inks for visualization. Isolated axon (**B**) without the CSPG treatment (control) and (**C**) with the CSPG treatment inside the axon compartments. Cells were stained with Calcein-AM for visualization. Scale bars: 50 µm.

In order to carry out six sets of independent localized biomolecular treatments with four replicates for each condition, fluidic isolation between the soma compartment and the axon compartments as well as fluidic isolation among 24 axon compartments is required. Fluidic isolation between the soma compartment and the axon compartments was achieved by generating fluidic level difference between the compartments that counters diffusion of locally treated biomolecules (Figure 6A,B). For the developed platform, culture medium is changed all together by a single aspiration at the exchange port via culture medium outflow channel. Although all axon compartments are connected via medium outflow channel, during replenishment, culture medium from one axon compartment cannot flow into any other axon compartments as demonstrated by the fluidic simulation results. In addition, culture medium inside the axon reservoirs and medium outflow channel is all aspirated out after the replenishment procedure (Figure 6B), thereby ruling out any potential risk of cross contamination among axon compartments. In order to demonstrate the fluidic isolation and the multiple localized biomolecular treatment capability, only the isolated axons connected to two reservoirs (blue and orange reservoirs in Figure 7A) were exposed to CSPG (5 μg/mL), a family of proteoglycan known to negatively regulate axon growth and cause retraction/degeneration of the established CNS axons. After 48 h, Calcein-AM staining of the axons inside the axon compartment revealed significant degeneration and fragmentation of the isolated axons inside the axon compartments treated with CSPG compared to the control (Figure 7B,C). This clearly demonstrates that the developed platform can indeed be used as a high-throughput compartmentalized neuron culture platform for screening potential drugs that promote axon growths.

## 4. Conclusions

A novel compartmentalized neuron culture platform that can be used for axon growth drug screening has been developed. The platform is composed of 24 axon compartment capable of performing six sets of independent biomolecular treatments with four replicates on a single device in parallel. The proposed fabrication method enabled to fabricate a PDMS device having 24 axon compartments, with two pre-defined through-holes, connected to the center soma compartment via shallow microchannel arrays to be prepared by a single PDMS softlithography process. Axons were successfully isolated inside the axon compartments and could be locally treated with different biomolecules for screening. In addition, the unique microfluidic configuration, validated by the simulation results and the localized CSPG treatment experiments, enabled culture medium in the 24 axon compartments to be replenished all together by a single aspiration step while maintaining fluidic isolation among axon compartments. We believe that the simple fabrication method and localized biomolecular treatment capability, combined with the simple culture medium replenishment feature will make this device a true high-throughput neuron culture microsystem for drug screening.

## Supplementary Materials

The following are available online at http://www.mdpi.com/2072-666X/7/7/114/s1, Figure S1: Detailed dimensions of (A) the reservoir layer, (B) the compartment layer, and (C) the substrate layer; Figure S2: Comparison of fluidic flow profiles between two different branch channel designs. (A) All branch channels have an identical dimensions (height: 500 μm, width: 1500 μm); (B) Platform with different dimension branch channels to compensate the fluidic resistance difference (as shown in Figure 4C). Similar fluidic flow was observed at each branch channel while the one with all same branch channel design showed uneven fluidic flow (most of flow was observed from the axon compartments closer to the culture medium exchange port). Note that the size of arrows is proportional to the flow speed of culture medium.

## Abbreviations

The following abbreviations are used in this manuscript:

CNS	Central nervous system
PNS	Peripheral nervous system
MAG	Myelin-associated glycoprotein
OMgp	Oligodendrocyte myelin glycoprotein
PDMS	Poly(dimethylsiloxane)
CNC	Computer numerically controlled
PMMA	Poly(methyl methacrylate)
AM	Acetoxymethyl
SEM	Scanning electron microscope
IPA	Isopropyl alcohol
DI water	Deionized water
E16	Embryonic day 16
DIV	Days in vitro
CSPG	Chondroitin sulfate proteoglycan
PBS	Phosphate buffered saline
FEM	Finite element method
