# Supplementary material for: A Microchip for High-Throughput Axon Growth Drug Screening
Source: Micromachines (Basel). 2016 Jul 7;7(7):114. doi: 10.3390/mi7070114 (PMC5137948; doi:10.3390/mi7070114)
Supplement: Supplementary file 1 [file micromachines-07-00114-s001.pdf]

Supplementary Materials: A Microchip for High-Throughput Axon Growth Drug Screening

Hyun Soo Kim, Sehoon Jeong, Chiwan Koo, Arum Han and Jaewon Park

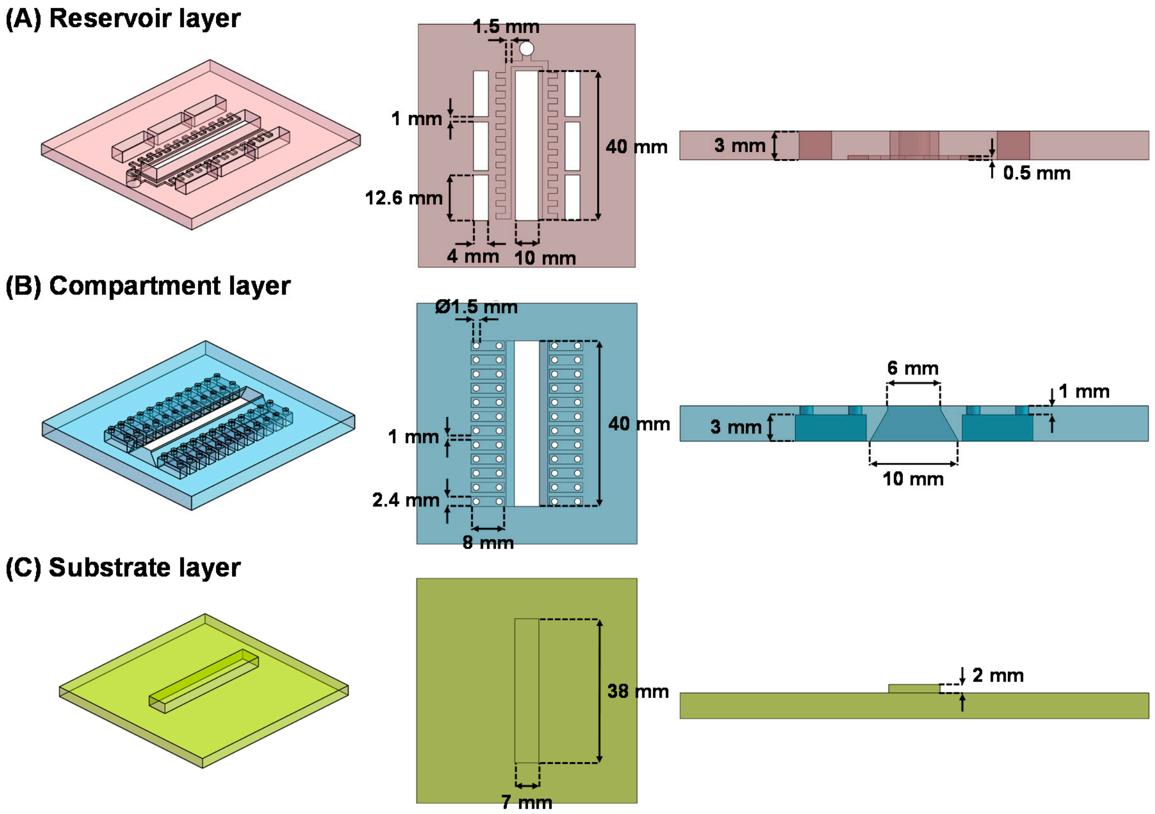

Figure S1. Detailed dimensions of (**A**) the reservoir layer, (**B**) the compartment layer, and (**C**) the substrate layer.

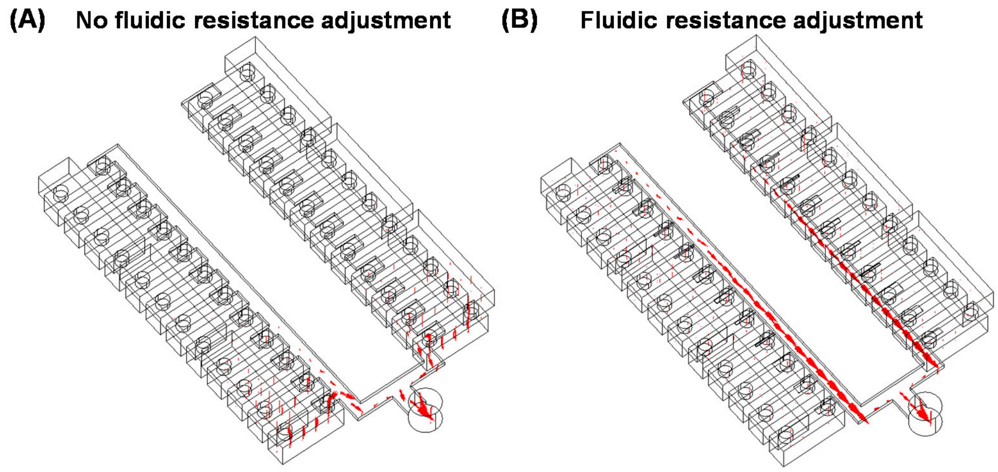

Figure S2. Comparison of fluidic flow profiles between two different branch channel designs: (**A**) all branch channels have an identical dimensions (height: 500 μm, width: 1500 μm); and (**B**) platform with different dimension branch channels to compensate the fluidic resistance difference (as shown in Figure 4C). Similar fluidic flow was observed at each branch channel while the one with all same branch channel design showed uneven fluidic flow (most of flow was observed from the axon compartments closer to the culture medium exchange port). Note that the size of arrows is proportional to the flow speed of culture medium.
